# P-1481. Short-Treatment with Ceftazidime-Avibactam for Carbapenemase-Producing Enterobacterales Bacteremia

**DOI:** 10.1093/ofid/ofae631.1651

**Published:** 2025-01-29

**Authors:** Fabián Herrera, María V Leone, Diego Torres, Rocío Rojas, Florencia Bues, Jimena Carlos, Elena Temporiti, Marcia Querci, Federico Nicola, Silvia Relloso, Jorgelina Smayevsky, Pablo E Bonvehi

**Affiliations:** Centro de Educación Médica e Investigaciones Clínicas, CEMIC, BUENOS AIRES, Ciudad Autonoma de Buenos Aires, Argentina; Hospital Universitario CEMIC, Buenos Aires, Ciudad Autonoma de Buenos Aires, Argentina; Centro de Educación Médica e Investigaciones Clínicas, CEMIC, BUENOS AIRES, Ciudad Autonoma de Buenos Aires, Argentina; Hospital Universitario CEMIC, Buenos Aires, Ciudad Autonoma de Buenos Aires, Argentina; Hospital Universitario CEMIC, Buenos Aires, Ciudad Autonoma de Buenos Aires, Argentina; Hospital Universitario CEMIC, Buenos Aires, Ciudad Autonoma de Buenos Aires, Argentina; Hospital Universitario CEMIC, Buenos Aires, Ciudad Autonoma de Buenos Aires, Argentina; Hospital Universitario CEMIC, Buenos Aires, Ciudad Autonoma de Buenos Aires, Argentina; Hospital Universitario CEMIC, Buenos Aires, Ciudad Autonoma de Buenos Aires, Argentina; Hospital Universitario CEMIC, Buenos Aires, Ciudad Autonoma de Buenos Aires, Argentina; Hospital Universitario CEMIC, Buenos Aires, Ciudad Autonoma de Buenos Aires, Argentina; Hospital Universitario CEMIC, Buenos Aires, Ciudad Autonoma de Buenos Aires, Argentina

## Abstract

**Background:**

Ceftazidime-avibactam (CA) and CA plus aztreonam (ATM) are the preferred treatment options for KPC and metallo-beta-lactamase (MBL) carbapenemase-producing Enterobacterales bacteremia (CPEB). However, data regarding short therapy are scarce.

**Methods:**

Prospective observational cohort study performed in a university hospital in Argentina between August 2018 and February 2024. All adult patients with CPEB who received adequate empirical treatment with CA or CA+ATM, with a median duration of 7 days (IQR: 7-8), with clinical response and source control of infection were included. They were followed for 30 days to evaluate mortality.

**Results:**

Twenty-two patients were included. They had a median age of 61 years (IQR: 49-70.5). The median APACHE II and Pitt scores were 18 (IQR: 13.25-24) and 2 (IQR: 1-4). Sixty-eight percent of the patients were immunocompromised, being hematological malignancies (45.45%), high-dose corticosteroids (40.9%) biological agents/anti-lymphocyte therapies (36.36%), and neutropenia (31.81%) the most frequent causes. Eighty-six percent had a clinical source of bacteremia; abdominal, central venous catheter, and urinary tract being the most common. *Klebsiella* spp. (72.72%) was the main etiological isolated microorganism, and the resistance mechanisms involved were KPC production (77.2%), MBL production (13.6%), and KPC+MBL production (9%). Recent colonization with KPC-CPE and MBL-CPE was detected in 68.1% and 9% of the patients. Septic shock at onset and intensive care unit requirement were 40.9% and 45.4% respectively. Empirical and definitive antibiotic therapy were combined in 66.6% and 9% of the patients. Thirty-day overall and infection-related mortality were 22.7% and 9%.

**Conclusion:**

This data showed that short-course antibiotic treatment with CA or CA+ATM for patients with CPEB who had clinical response with source control of infection might be adequate.

**Disclosures:**

**Diego Torres, Medical Doctor**, Gador: Honoraria|GSK: Advisor/Consultant|Khight Therapeutics: Honoraria|MSD: Honoraria

